# Supplementary material for: The Evolution and Characterization of the RNA Interference Pathways in Lophotrochozoa
Source: Genome Biol Evol. 2024 May 7;16(5):evae098. doi: 10.1093/gbe/evae098 (PMC11114477; doi:10.1093/gbe/evae098)
Supplement: evae098_Supplementary_Data [file evae098_supplementary_data.zip › Supplementary_materials.zip!/selected_miRNAs/NoGe_selected_miRNAs/novel_miRNA_families_pdf_files/NMRB01002591.1_Notospermus_geniculatus_strain_Ushimado_scaffold2591,_whole_genome_shotgun_sequence_39377.pdf]

| | | |
|---|---|---|
| Provisional ID | : | NMRB01002591.1_Notospermus_geniculatus_strain_Ushimado_scaffold2591,_whole_genome_shotgun_sequence_39377 |
| Score total | : | 1721 |
| Score for star read(s) | : | 3.9 |
| Score for read counts | : | 1712.1 |
| Score for mfe | : | 1.9 |
| Score for randfold | : | 0 |
| Score for cons. seed | : | 3 |
| Total read count | : | 3370 |
| Mature read count | : | 3164 |
| Loop read count | : | 0 |
| Star read count | : | 206 |

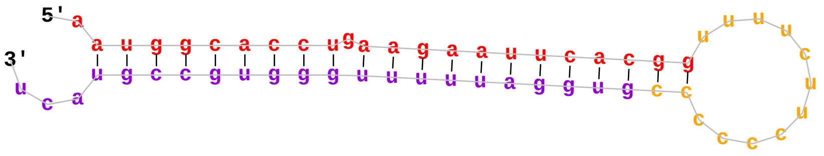


Genome coordinates: NMRB01002591.1 20682-20740

pre-miRNA sequence:
AATGGCACCTGAAGAATTCACGGAATGGCACCTGAAGAATTCACGGTTTTCTTCCCCCCGTGGATTTTTGGGTGCCGTACT

Mature sequence: AATGGCACCTGAAGAATTCACGG

BLAST results for the pre-miRNA sequence against the *Lineus longissimus* genome assembly:

| **Scaffold** | **Start** | **Stop** | **Identity** | **e-value** |
|---|---|---|---|---|
| OU342999.1 1 | 1849517 | 1849539 | 100.000 | 0.001 |

```
Nge_novel_miRNA                    AATGGCACCTGAAGAATTCACGG
Llo_genome                         AATGGCACCTGAAGAATTCACGG
```
